# Amount of fibroglandular tissue FGT and background parenchymal enhancement BPE in relation to breast cancer risk and false positives in a breast MRI screening program: A retrospective cohort study

**DOI:** 10.1007/s00330-019-06020-2

**Published:** 2019-02-22

**Authors:** Suzan Vreemann, Mehmet U. Dalmis, Peter Bult, Nico Karssemeijer, Mireille J. M. Broeders, Albert Gubern-Mérida, Ritse M. Mann

**Affiliations:** 1 Department of Radiology and Nuclear Medicine, Radboud University Medical Center, Geert Grooteplein 10, route 766, 6525 GA Nijmegen, the Netherlands; 2 Department of Pathology, Radboud University Medical Center, Nijmegen, the Netherlands; 3 Department for Health Evidence, Radboud University Medical Center, Nijmegen, the Netherlands

**Keywords:** Breast, Magnetic resonance imaging, Breast neoplasms, Risk factors

## Abstract

**Objectives:**

The purpose of this study is to evaluate the predictive value of the amount of fibroglandular tissue (FGT) and background parenchymal enhancement (BPE), measured at baseline on breast MRI, for breast cancer development and risk of false-positive findings in women at increased risk for breast cancer.

**Methods:**

Negative baseline MRI scans of 1533 women participating in a screening program for women at increased risk for breast cancer between January 1, 2003, and January 1, 2014, were selected. Automated tools based on deep learning were used to obtain quantitative measures of FGT and BPE. Logistic regression using forward selection was used to assess relationships between FGT, BPE, cancer detection, false-positive recall, and false-positive biopsy.

**Results:**

Sixty cancers were detected in follow-up. FGT was only associated to short-term cancer risk; BPE was not associated with cancer risk. High FGT and BPE did lead to more false-positive recalls at baseline (OR 1.259, *p* = 0.050, and OR 1.475, *p* = 0.003) and to more frequent false-positive biopsies at baseline (OR 1.315, *p* = 0.049, and OR 1.807, *p* = 0.002), but were not predictive for false-positive findings in subsequent screening rounds.

**Conclusions:**

FGT and BPE, measured on baseline MRI, are not predictive for overall breast cancer development in women at increased risk. High FGT and BPE lead to more false-positive findings at baseline.

**Key Points:**

*• Amount of fibroglandular tissue is only predictive for short-term breast cancer risk in women at increased risk.*

*• Background parenchymal enhancement measured on baseline MRI is not predictive for breast cancer development in women at increased risk.*

*• High amount of fibroglandular tissue and background parenchymal enhancement lead to more false-positive findings at baseline MRI.*

**Electronic supplementary material:**

The online version of this article (10.1007/s00330-019-06020-2) contains supplementary material, which is available to authorized users.

## Introduction

Women at increased risk of breast cancer (≥ 20–25% lifetime risk) are eligible for intensified screening programs, including a yearly breast magnetic resonance imaging (MRI) study. Depending on the underlying risk factors, MRIs may be performed on an annual basis from the age of 25 (in *BRCA* mutation carriers) [1, 2]. Women with a hereditary germline mutation and women with a history of radiation therapy to the chest at a young age are eligible to these programs. For other women, risk-prediction tools are used to determine whether women are at increased risk and thus eligible for MRI screening. The current risk-prediction tools rely mainly on personal factors, such as family history, age, and race [3, 4]. However, recent studies show that additional independent risk factors, including imaging biomarkers, might increase the predictive power of risk prediction.

Mammographic breast density (BD), for example, correlates to breast cancer risk in the general female population and in *BRCA* mutation carriers [5, 6]. Consequently, a number of studies recommend adding BD to the available risk prediction tools [7–10].

The increased use of breast MRI allows for evaluation of additional risk factors to improve current risk prediction tools. Recent publications indicate that the amount of fibroglandular tissue (FGT) and/or background parenchymal enhancement (BPE) measured on breast MRI may be useful to predict breast cancer risk in women undergoing breast MRI [11–13], although results have to be interpreted with caution [14].

While in breast MRI all normal FGT enhances after contrast injection, the strength and speed of enhancement is dependent on variations in hormone levels, as determined by menstrual cycle phase, menopausal status, tamoxifen therapy, and hormone replacement therapy [15–17]. Studies of King et al and Dontchos et al [11, 12] showed that higher amounts of BPE in the contralateral breast increase the risk of breast cancer diagnosis. Their results thus suggest that BPE might be used for the prediction of breast cancer risk. Unfortunately, both studies evaluated BPE at time of breast cancer detection and are therefore unable to document its predictive value for future breast cancer occurrence.

A further problem is that visual rating of BPE on a four-point scale (minimal < 25%, mild 25–50%, moderate 50–75%, and marked 75–100%), as used in studies so far, suffers from high interrater variability [18]. This limits its value for risk prediction. Analogue to systems currently in use to automatically estimate BD on digital mammograms, automated tools to assess FGT and BPE may reduce interrater variability and possibly provide more robust parameters for risk stratification.

The purpose of this study is to study whether FGT and BPE, as computed on a cancer-free baseline MRI scan using an automated tool, are predictors of future breast cancer in a breast MRI screening program. Furthermore, we evaluate whether FGT and BPE predict false-positive findings.

## Materials and methods

This retrospective cohort study was approved by our local institutional review board, and the requirement for informed consent was waived.

### Screening program

The breast cancer screening program for women with a lifetime risk of ≥ 20–25% at our institution consists of annual breast MRI and mammography [1, 19]. In *BRCA* mutation carriers, the screening regimen starts with breast MRI only at age 25. Mammography is added from age 30. Others start screening with both modalities at age 35 or 40, depending on the age at which relatives developed breast cancer. The first MRI scan performed for screening is hereafter referred to as “baseline” MRI.

### Case selection

**Fig. 1:**
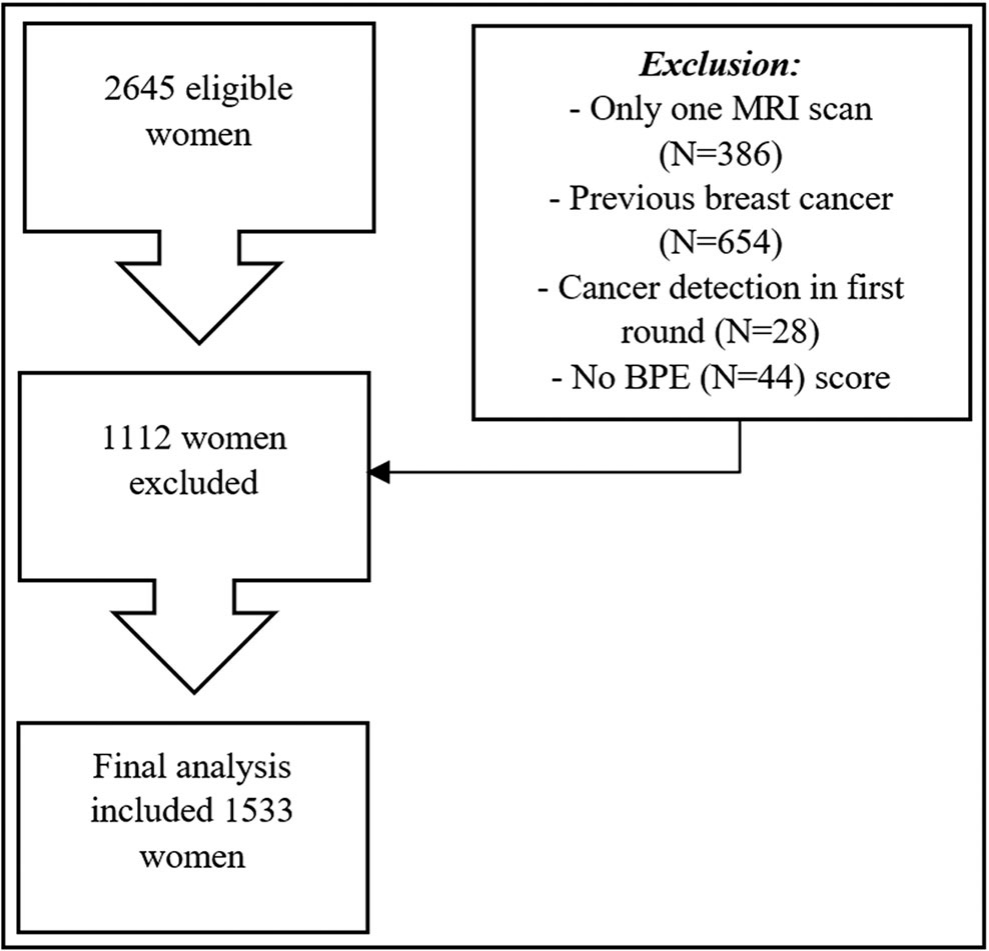
Flow diagram of the case selection procedure. BPE, background parenchymal enhancement; FGT, amount of fibroglandular tissue

The local database was searched to identify all patients who underwent breast MRI screening between January 1, 2003, and January 1, 2014. The case selection process is presented in Fig. 1. Women of any age were included when they underwent at least two breast MRI examinations for screening in this period. We recorded for each patient whether a *BRCA* mutation was present and whether and when a risk-reducing salpingo-oophorectomy (RRSO) was performed. Women in whom a cancer was detected at baseline MRI or within 6 months thereafter, women with a prior history of breast cancer, and women in whom automated assessment of BPE failed were excluded. We did not exclude women who had a false-positive finding in the first round of screening.

### Ground truth

Normal or benign screening examinations were confirmed by at least one year of clinical follow-up and regarded as true negative when no cancer was detected before the subsequent screening examination. When no biopsy was indicated at short-term follow-up, at least one year of clinical follow-up was required to confirm benignity. Biopsied lesions were identified by a cross-computer search with our pathology records. We subsequently analyzed if the biopsy was performed based on screening findings or for other reasons (e.g., symptoms).

### Image acquisition

MRI protocols varied over time [20]. Dynamic contrast-enhanced breast MRI acquisitions were performed on either a 1.5- or 3-T Siemens scanner using a dedicated bilateral breast coil. Patients were placed in prone position. A transverse or coronal three-dimensional T1-weighted gradient-echo dynamic sequence was performed before contrast agent administration followed by four or five post-contrast sequences. The first time point was acquired before intravenous agent injection and the following time points after contrast agent injection. The gadolinium chelates were administered at a dose of 0.1 mmol/kg or 0.2 mmol/kg using a power injector (Medrad), followed by a saline flush.

### Imaging interpretation

**Fig. 2:**
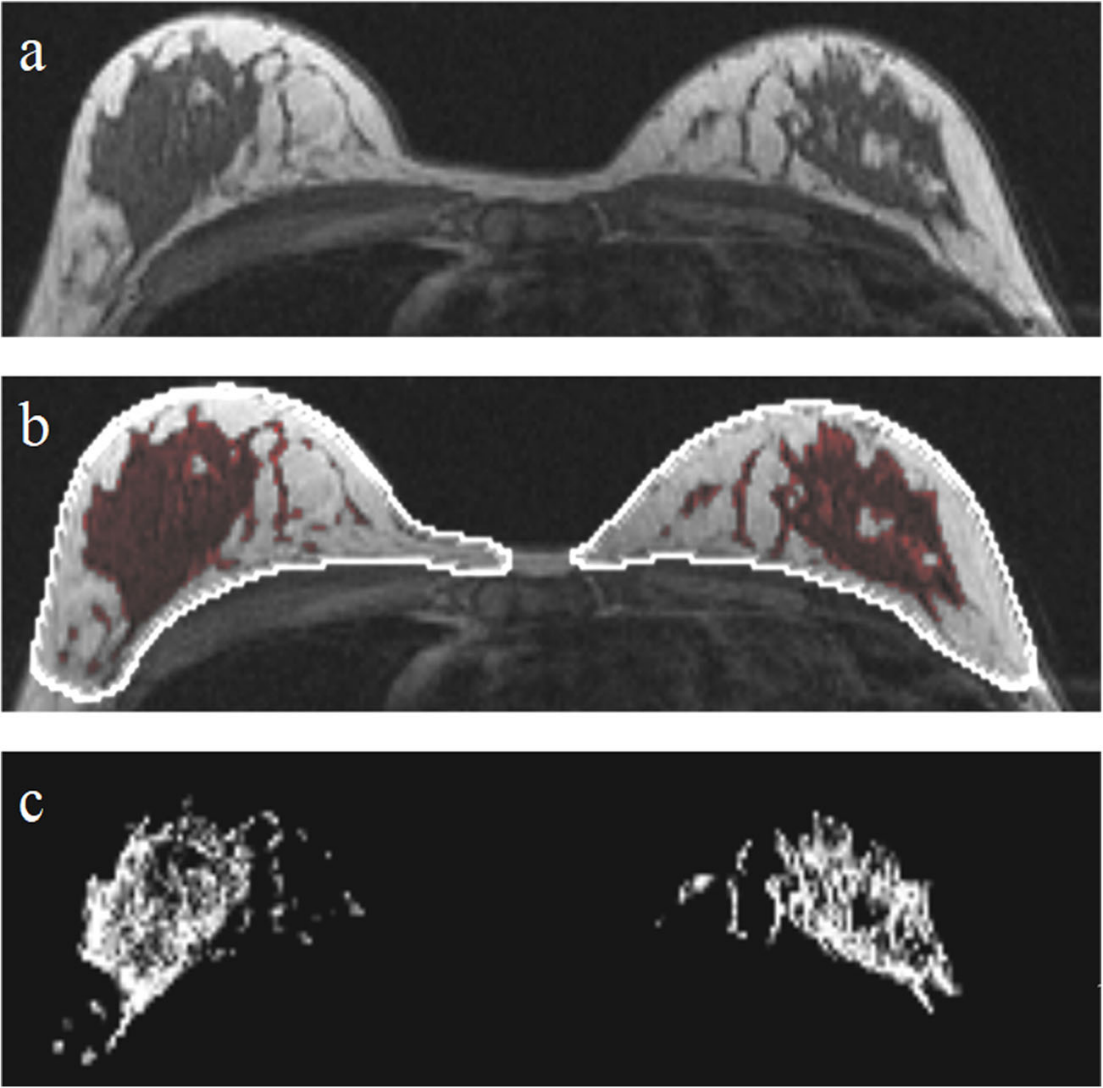
An example of the steps of the automated tool to determine the amount of fibroglandular tissue (FGT) and background parenchymal enhancement (BPE). First, the original image (**a**), then the breasts and parenchymal tissue (**b**) are segmented, and finally, relative enhancement values are computed for the segmented FGT volumes (**c**). BPE values are extracted from the enhancing voxels within the parenchymal tissue, based on these relative enhancement values

Automatic tools were used to objectively calculate percentages of FGT and BPE on breast MRI volumes. Breast and FGT were segmented on native T1-weighted pre-contrast acquisitions using a deep-learning-based method as described and validated in [20]. The fraction of FGT was calculated as the segmented volume of FGT divided by the total breast volume. BPE relative enhancement values were computed using the pre-contrast and the first post-contrast T1-weighted acquisition after motion correction [21], according to the ACR guidelines [22, 23]. The fraction of BPE is expressed relative to the volume of FGT, where an FGT voxel is considered to enhance if it has a relative enhancement value higher than 10%, which correlates best to radiologist rates according to Dalmis et al [24]. Figure 2 shows an example of automated computations of FGT and BPE. Final FGT and BPE measurements were the result of averaging over the two breasts of each woman. To verify whether correlations change when investigating different cut-off values, we performed the same analyses on 20%, 30%, 40%, and 50% relative enhancement values (Supplementary Table 2).

### Data analysis

Women who developed cancer were identified by linkage of our data to the Netherlands Comprehensive Cancer Organisation. False-positive MRI examinations were defined as examinations that led to recall in women in whom no breast cancer was detected. False-positive recalls (FPR) include all women who were recalled (with or without performance of biopsy). False-positive biopsies (FPB) only include women for whom the recall led to biopsy. We separated FPR and FPB in first rounds from those that occurred in subsequent screening rounds.

To investigate the influence of time between the baseline scan and cancer development, we performed a subgroup analysis in patients who developed breast cancer within 2 years after the baseline MRI scan.

### Statistical analysis

Incomplete data was assumed to be missing at random and was excluded. Descriptive statistics were prepared with the use of contingency-table analyses for categorical data and Fisher’s exact tests. The 95% confidence intervals (95% CI) for proportions were estimated using the *Z* test for single proportions. Continuous data were compared with the Student’s *t* test or Pearson correlation coefficient (*r*) when normally distributed; otherwise, Mann-Whitney *U* tests were used. Bootstrapping (*N* = 1000) was used to calculate 95% CI. To increase statistical power, FGT and BPE were dichotomized into two categories based on the optimal categories in ROC analysis (0–50th percentile and 50–100th percentile). A binary logistic regression model was constructed to find independent predictors for breast cancer or false positives. Separate and combined models were performed for FGT and BPE. Inclusion of variables in the model was based on existing knowledge of risk factors for breast cancer and/or false positives (covariates: age and *BRCA* status). Non-linear effects were evaluated using Box-Tidwell tests and when needed transformations were performed. The value of predictors was assessed by using forward feature selection (using a liberal probability-to-enter of 0.1). Interactions between predictors were evaluated in the final models by including interaction terms along with the main-effect terms. The final model was bootstrapped (*N* = 1000). Shrinkage using the heuristic method was applied to account for over-optimism [25]. Odds ratios (OR) were used to report on the relative odds of occurrence of the outcome (future cancer, or false-positive result), where OR = 1, the predictor does not affect odds of outcome; OR > 1, the predictor is associated with higher odds of outcome; and OR < 1, the predictor is associated with lower odds of outcome. All statistical tests were two-sided. *p* ≤ 0.05 was considered significant. All statistics were performed in SPSS (v.22, SPSS Inc.).

## Results

### Population

The final analysis evaluated baseline breast MRI scans of 1533 women, including 573 (37.4%) *BRCA* mutation carriers (Supplementary Table 1). Patient selection and exclusion are shown in Fig. 1. The median age at baseline was 41 years (37 years for *BRCA* mutation carriers and 44 years for others). In 60 (3.9%) women, cancer was identified after a negative baseline scan. Forty-five (75%) cancers were screen-detected cancers, six (10%) were interval cancers, and nine (15%) were detected in prophylactic mastectomies. Forty-three (71.7%) cancers were invasive and 17 (28.3%) were ductal carcinoma in situ (DCIS) only. The median time between the negative baseline scan and cancer detection was 3 years (two in *BRCA* patients, three in others). Of the 573 *BRCA* mutation carriers, 103 (18%) women had a RRSO prior to the first screening round, while 227 underwent RRSO after the first screening round.

Three hundred thirty-seven (22.0%) women had a false-positive recall. Seventy-three (21.7%) of these women were recalled based upon mammography findings. Two hundred sixty-four (78.3%) women had at least one false-positive recall based on the MRI exam (total, 286 recalls on MRI), and 203 (13.2%) women had at least one false-positive biopsy due to MRI findings (total, 217 biopsies). Median FGT measured on MRI was 12.7% (interquartile range (IQR), 18.9%), and median BPE was 67.7% (IQR, 27.6%). Tables 1, 2, and 3 have a more detailed presentation of the population characteristics.

**Table 1:** Baseline patient characteristics

	Total cohort (*N* = 1533)	Developed cancer	
		Yes (*N* = 60)	No (*N* = 1473)
Age in years; median^$^ (IQR)	41 (17.0)	40 (13.0)	42 (17.0)
BRCA mutation carriers; *N* (fraction*)	573, 0.37	41, 0.68	532, 0.36
FGT in percentage; median^$^ (IQR)	12.7 (18.9)	11.6 (19.8)	12.7 (18.7)
BPE in percentage; median^$^ (IQR)	67.7 (27.6)	71.3 (30.4)	67.6 (27.6)
Cancer; *N* (fraction*)	60, 0.04	60 (N/A)	N/A
Age at cancer detection; median^$^ (IQR)	42 (15.0)	42 (15.0)	N/A
False-positive recall overall; *N* (fraction*)	337, 0.22	19, 0.32	318, 0.22
Age at recall; median^$^ (IQR)	42 (15.0)	40 (18.0)	42 (15.0)
False-positive recall MRI; *N* (fraction*)	264, 0.17	16, 0.27	248, 0.17
Age at recall; median^$^ (IQR)	40 (15.0)	39 (16.8)	40 (15.0)
False-positive biopsy overall; *N* (fraction*)	221, 0.14	12, 0.20	209, 0.14
Age at biopsy; median^$^ (IQR)	41 (14.5)	39 (16.5)	41 (14.5)
False-positive biopsy MRI; *N* (fraction*)	203, 0.13	11, 0.18	192, 0.13
Age at biopsy; median^$^ (IQR)	40 (15.0)	38 (18.0)	40 (15.0)
RRSO; *N* (fraction*)	103, 0.07	5, 0.08	98, 0.07

*N/A* not applicable, *BPE* background parenchymal enhancement, *FGT* amount of fibroglandular tissue, *IQR* the difference between the 75th and 25th percentiles, *RRSO* risk-reducing salpingo-oophorectomy

^*^Fraction of positive cases compared to the complete cohort

^$^Tested on normality using the Kolmogorov-Smirnov test

**Table 2:** Baseline characteristics of *BRCA* mutation carriers

	Total cohort (*N* = 573)	Developed cancer	
		Yes (*N* = 41)	No (*N* = 532)
Age in years; median^$^ (IQR)	37 (17)	41 (14.5)	37 (18)
FGT in percentage; median^$^ (IQR)	9.3 (14.5)	10.7 (16.7)	9.3 (14.5)
BPE in percentage; median^$^ (IQR)	65.6 (26.7)	71.2 (33.3)	65.1 (26.1)
Cancer; *N* (fraction*)	41, 0.07	41, 1.00	0, 0.00
Age at cancer detection; median^$^ (IQR)	42 (14.5)	42 (14.5)	N/A
False-positive recall overall; *N* (fraction*)	118, 0.21	12, 0.29	106, 0.20
Age at recall; median^$^ (IQR)	38.5 (15)	38 (19.5)	39 (14.3)
False-positive recall MRI; *N* (fraction*)	97, 0.17	10, 0.24	87, 0.16
Age at recall; median^$^ (IQR)	38 (14.5)	38 (18.75)	39 (14)
False-positive biopsy overall; *N* (fraction*)	80, 0.14	7, 0.17	73, 0.14
Age at biopsy; median^$^ (IQR)	38 (14)	34 (11)	39 (14.5)
False-positive biopsy MRI; *N* (fraction*)	72, 0.13	7, 0.17	65, 0.12
Age at biopsy; median^$^ (IQR)	38 (15.3)	34 (11)	38 (15.5)
RRSO; *N* (fraction*)	103, 0.18	5, 0.12	98, 0.18

*N/A* not applicable, *BPE* background parenchymal enhancement, *FGT* amount of fibroglandular tissue, *IQR* the difference between the 75th and 25th percentiles, *RRSO* risk-reducing salpingo-oophorectomy

^*^Fraction of positive cases

^$^Tested on normality using the Kolmogorov-Smirnov test

**Table 3:** Baseline characteristics of others at increased risk

	Total cohort (*N* = 960)	Developed cancer	
		Yes (*N* = 19)	No (*N* = 941)
Age in years; median^$^ (IQR)	44 (15)	40 (11)	44 (15)
FGT in percentage; median^$^ (IQR)	14.9 (20.7)	20.8 (20.5)	14.8 (20.6)
BPE in percentage; median^$^ (IQR)	69.0 (27.6)	73.4 (29.1)	69.0 (27.6)
Cancer; *N* (fraction*)	19, 0.02	19, 1.00	N/A
Age at cancer detection; median^$^ (IQR)	43 (16)	43 (16)	N/A
False-positive recall overall; *N* (fraction*)	219, 0.23	7, 0.37	212, 0.23
Age at recall; median^$^ (IQR)	43 (14)	46 (15)	43 (14)
False-positive recall MRI; *N* (fraction*)	167, 0.23	6, 0.32	161, 0.17
Age at recall; median^$^ (IQR)	42 (15)	47.5 (17.8)	42 (14)
False-positive biopsy overall; *N* (fraction*)	141, 0.15	5, 0.26	136, 0.14
Age at biopsy; median^$^ (IQR)	43 (14)	46 (10)	43 (14)
False-positive biopsy MRI; *N* (fraction*)	131, 0.14	4, 0.21	127, 0.13
Age at biopsy; median^$^ (IQR)	42 (14)	47.5 (12.5)	42 (14)
RRSO; *N* (fraction*)	0, 0.00	0, 0.00	0, 0.00

*N/A* not applicable, *BPE* background parenchymal enhancement, *FGT* amount of fibroglandular tissue, *IQR* the difference between the 75th and 25th percentiles, *RRSO* risk-reducing salpingo-oophorectomy

^*^Fraction of positive cases

^$^Tested on normality using the Kolmogorov-Smirnov test

**Fig. 3:**
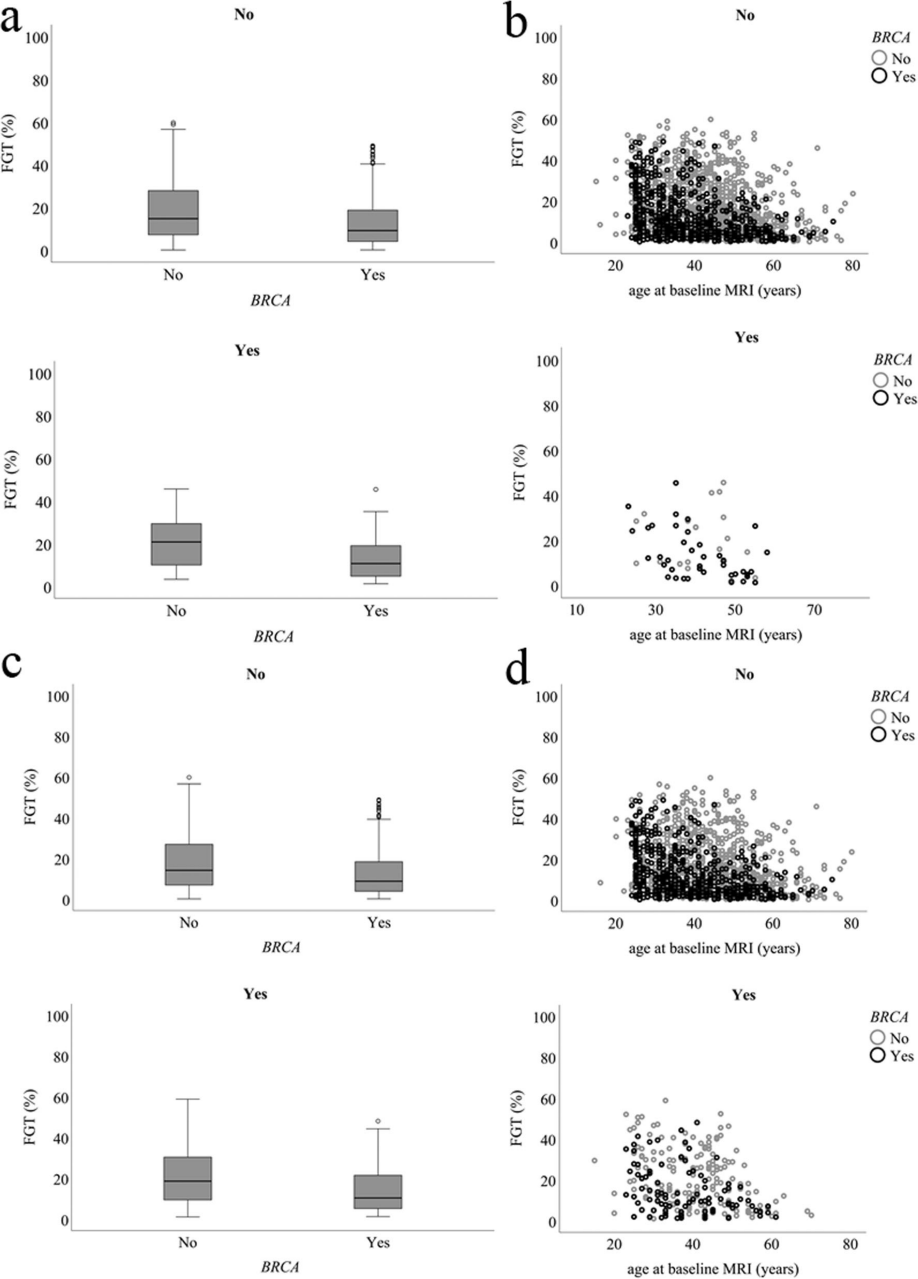
Distribution of the amount of fibroglandular tissue (FGT) for women with a *BRCA* mutation and women without a *BRCA* mutation. In **a**, box plots show the lowest and highest FGT values (outermost horizontal lines), median FGT (central horizontal line), and interquartile range (top and bottom borders of the box) for breast cancer (no/yes). Scatter plots show the association of FGT to breast cancer occurrence (no/yes) and age at baseline MRI (**b**). In **c**, boxplots are shown for false-positive recall occurrence (no/yes), and in **d**, scatterplots show the association of FGT to false-positive recall occurrence

**Fig. 4:**
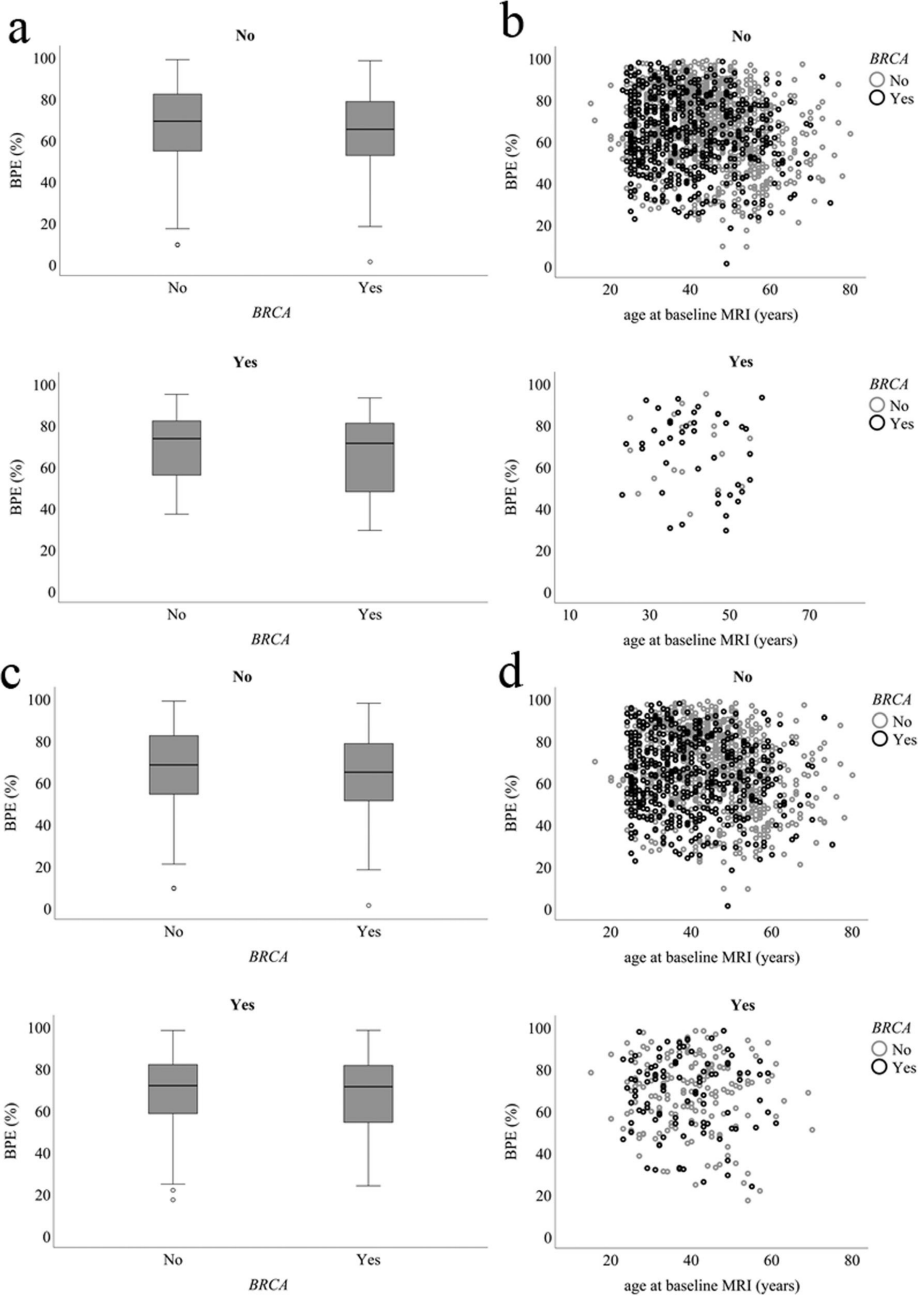
Distribution of the background parenchymal enhancement (BPE) for women with a *BRCA* mutation and women without a *BRCA* mutation. In **a**, box plots show the lowest and highest BPE values (outermost horizontal lines), median BPE (central horizontal line), and interquartile range (top and bottom borders of the box) for breast cancer (no/yes). Scatter plots show the association of BPE to breast cancer occurrence (no/yes) and age at baseline MRI (**b**). In **c**, boxplots are shown for false-positive recall occurrence (no/yes), and in **d**, scatterplots show the association of BPE to false-positive recall occurrence

In univariate analysis, a significant association between FGT and *BRCA* status was found in both percentages (*p* = 0.001) and the dichotomous scores (*p* < 0.001). *BRCA* mutation carriers had lower FGT scores than others. The *BRCA* mutation carriers had a lower age at the baseline scan (median age of 37 for *BRCA* mutation carriers versus 44 for others, *p* < 0.001). A similar association was found between the percentage of BPE and *BRCA* status (*p* = 0.005), as *BRCA* mutation carriers had significantly lower BPE scores. When dichotomizing BPE, this remained significant (*p* = 0.020). FGT and BPE were negatively correlated to age (*r* = − 0.289 and *r* = − 0.129, *p* < 0.001), also when using dichotomous values (*p* ≤ 0.007). In *BRCA* mutation carriers, coefficients were *r* = − 0.418 (*p* < 0.001) and *r* = − 0.132 (*p* = 0.002), respectively, and in women without a *BRCA* mutation, *r* = − 0.307 (*p* < 0.001) and *r* = − 0.152 (*p* < 0.001). FGT and BPE were not correlated (*p* = 0.879). Plots of the univariate analysis are presented in Figs. 3 and 4. In *BRCA* mutation carriers, BPE was not associated with a history of RRSO (*p* = 0.886, Table 2).

### Cancer prediction models

In univariate analysis, FGT was not associated with breast cancer for both discrete (*p* = 0.768) and dichotomous values (*p* = 0.511). In regression analysis, FGT was not considered an independent risk factor for breast cancer; only *BRCA* status was (OR, 3.615; *p* = 0.001). Likewise, percentages and dichotomized BPE scores of the baseline MRI scan were not associated with breast cancer (*p* = 0.625 and *p* = 0.236). In regression analysis, adjusting for the only significant risk factor (*BRCA* status), BPE was also no significant predictor of cancer (*p* = 0.112). When evaluating both FGT and BPE, both were not significantly associated with breast cancer risk (*p* = 0.824 and *p* = 0.112). Also in the subgroup of the *BRCA* and non-*BRCA* mutation carriers only, BPE and FGT were not associated to breast cancer.

The subgroup analysis on cancers developed in the first two years after baseline MRI scan included 17 cancers and a total of 1499 baseline MRI scans. In univariate and multivariate analyses, continuous BPE was still not associated to breast cancer (*p* = 0.302), but FGT was (*p* = 0.030). Dichotomizing BPE (*p* = 0.234) and FGT (*p* = 0.012) did not change results. In regression analysis, FGT was associated to breast cancer (*p* = 0.036), but BPE was not (*p* = 0.137). Details of predictors can be found in Table 4.

**Table 4:** Regression coefficients and odds ratios for the prognostic cancer prediction model

Model	Predictor	*p* value	Included in final model	*β*	OR	Shrinkage factor
				(95% CI)	(95% CI)	
Overall population						
Cancer-FGT	BRCA*	< 0.001	x	1.285 (0.762 to 1.872)	3.615 (2.143 to 6.501)	0.96
	Age	0.930	–			
	FGT	0.511	–			
Cancer-BPE	BRCA*	< 0.001	x	1.285 (0.769 to 1.875)	3.615 (2.158 to 6.521)	0.96
	Age	0.930	–			
	BPE	0.236	–			
Subgroup *BRCA*						
Cancer-FGT	Age	0.330	–	N/A		
	FGT	0.936	–			
Cancer-BPE	Age	0.330	–	N/A		
	BPE	0.106	–			
Subgroup non-*BRCA*						
Cancer-FGT	Age	0.126	–	N/A		
	FGT	0.621	–			
Cancer-BPE	Age	0.126	–	N/A		
	BPE	0.641	–			
Subgroup cancer within 2 years after baseline MRI scan						
Cancer-FGT	BRCA*	0.003	x	1.707 (0.696 to 15.557) − 1.078 (− 15.032 to − 0.076)	5.512 (2.006 to 5.706 · 10^6^) 0.340 (2.963 · 10^−7^ to 0.927)	0.90
	Age	0.507	–			
	FGT	0.036	x			
Cancer-BPE	BRCA*		x	1.961 (0.928 to 16.418)	7.106 (2.529 to 1.350 · 10^7^)	0.93
	Age	0.189	–			
	BPE	0.137	–			

For every model, different shrinkage factors were used; shrunk *β* and OR are presented

*β* standardized coefficients, *OR* odds ratio, *CI* confidence interval, *FGT* amount of fibroglandular tissue, *BPE* background parenchymal enhancement, *N/A* not applicable

^*^BRCA = 0 is reference category

### FPR models

When investigating the first-round results alone (diagnostic model), both FGT and BPE were correlated to higher FPR (OR, 1.259; *p* = 0.050, and OR, 1.475; *p* = 0.003, respectively). For subsequent rounds (prognostic model), higher FGT at baseline was still significantly related to higher FPR in both continuous and dichotomized values (*p* ≤ 0.029). BPE, however, was not related to FPR (*p* ≥ 0.818) in univariate analysis. In regression analysis, only age remained as related factor to FPR in follow-up (OR, 0.955, *p* = 0.001; Table 5).

**Table 5:** Regression coefficients and odds ratios for the effect on current and subsequent MRI scans on false-positive findings

Model	Predictor	*p* value	Included in final model	*β*	OR	Shrinkage factor
				(95% CI)	(95% CI)	
Diagnostic model for false-positive findings (current MRI scans)						
FPR-FGT	*BRCA*	0.771	–			
	Age	0.167	–			
	FGT*	0.050	x	0.230 (0.012 to 0.451)	1.259 (1.012 to 1.569)	0.74
FPR-BPE	*BRCA*	0.612	–			
	Age	0.102	–			
	BPE*	0.003	x	0.389 (0.120 to 0.666)	1.475 (1.128 to 1.946)	0.87
FPR-FGT and BPE	*BRCA*	0.894	–			
	Age	0.229	–			
	FGT*	0.072	x	0.251 (− 0.013 to 0.535)	1.285 (0.987 to 1.707)	0.83
	BPE*	0.005	x	0.366 (0.111 to 0.625)	1.442 (1.118 to 1.868)	
FPB-FGT	*BRCA*	0.350	–			
	Age	0.496	–			
	FGT*	0.049	x	0.274 (0.022 to 0.568)	1.315 (1.022 to 1.765)	0.74
FPB-BPE	*BRCA*	0.269	–			
	Age	0.362	–			
	BPE*	0.002	x	0.592 (0.253 to 0.957)	1.807 (1.288 to 2.605)	0.91
FPB-FGT and BPE	*BRCA*	0.453	–			
	Age	0.651	–			
	FGT*	0.064	x	0.312 (− 0.012 to 0.677)	1.367 (0.988 to 1.968)	0.87
	BPE*	0.002	x	0.559 (0.218 to 0.911)	1.750 (1.243 to 2.487)	
Prognostic model for false-positive findings						
FPR-FGT	*BRCA*	0.773	–	− 0.047 (− 0.069 to − 0.026)	0.955 (0.933 to 0.975)	0.95
	Age	0.001	x			
	FGT	0.224	–			
FPR-BPE	*BRCA*	0.773	–	− 0.047 (− 0.069 to − 0.026)	0.955 (0.933 to 0.975)	0.95
	Age	0.001	x			
	BPE	0.932	–			
FPB-FGT	*BRCA*	0.892	–	− 0.051 (− 0.081 to − 0.026)	0.951 (0.922 to 0.974)	0.94
	Age	0.001	x			
	FGT	0.557	–			
FPB-BPE	*BRCA*	0.892	–	− 0.051 (− 0.081 to − 0.026)	0.951 (0.922 to 0.974)	0.94
	Age	0.001	x			
	BPE	0.572	–			

For every model, different shrinkage factors were used; shrunk *β* and OR are presented

*β* standardized coefficients, *OR* odds ratio, *CI* confidence interval, *FGT* amount of fibroglandular tissue, *BPE* background parenchymal enhancement

^*^FGT and BPE = low is reference category

### FPB models

When only investigating the first round (diagnostic model), both FGT and BPE were correlated to higher FPB (OR, 1.315 (*p* = 0.049) and OR, 1.807 (*p* = 0.002), respectively).When excluding the FPB in the first round (prognostic model), FGT and BPE were both not related to FPB (*p* ≥ 0.066) in univariate analysis. Regression analysis showed that age was negatively related to FPB in follow-up (*p* = 0.001, Table 5).

No interaction terms were found to be significant in any of the prediction models. In addition, changing levels of BPE cutoffs did not change any of the conclusions for both the cancer- and false-positive prediction models (Supplementary Tables 2 and 3).

## Discussion

The purpose of this study was to investigate the predictive value of the amount of fibroglandular tissue (FGT) and background parenchymal enhancement (BPE) in predicting breast cancer risk in a population at increased risk of developing breast cancer. Additionally, the effect of FGT and BPE on false-positive recalls and biopsies was investigated. Our results show that neither FGT nor BPE at baseline was associated with the overall risk of developing breast cancer. However, in subgroup analysis only evaluating the cancers detected in the first 2 years after the baseline MRI scan, we found an association with FGT. Both higher FGT and BPE did lead to higher odds ratios for false-positive findings in the baseline examination. We did not observe any predictive value of FGT or BPE for FPR or FPB in subsequent screening rounds.

It has already been well established that mammographic breast density (BD) impairs mammographic sensitivity [26]. In an average-risk population, BD is also known to correlate with breast cancer risk [5]. In line with the studies from Dontchos et al [11] and Passaperuma et al [27] who reported that neither mammographic BD nor FGT on MRI were predictive of breast cancer risk in women at increased risk, we did not observe an overall correlation between FGT and the development of breast cancer in our high-risk cohort. However, Mitchell et al [6] reported contradictory results. In their study, it was suggested that high BD in *BRCA* mutation carriers increased the risk of breast cancer, with a relative risk similar to that observed in the general population. In our subgroup analysis, we found that FGT might be associated with early cancer development, and as FGT and BPE may change due to normal hormonal changes, it might be logical that the predictive potential in the longer run is relatively limited in this generally young population. However, as this analysis included small cancer numbers and results showed extremely large confidence intervals, these results need to be evaluated with caution. It should be noted that the differences between those studies and our findings may at least partly be explained by the automated volumetric FGT estimation in our study, which provides a different representation of the FGT than visually inspected BD in mammography, albeit previous studies showed a clear correlation between these measurements [28], and the applied method proved to be robust to variations in MRI acquisitions and breast density categories [19]. It may also be related to the limited sample size in our study and other studies published thus far.

Current clinical practice is shifting towards personalized screening, making risk prediction tools increasingly important. Recent case-control studies have shown that BPE might be predictive of breast cancer risk [11, 12], although contradictory results exist for non-high-risk women [29]. However, in these studies, the BPE scores of the healthy breast (partly for [11]) in breast cancer patients were compared to BPE scores in healthy controls. The current study, in which BPE before cancer development is evaluated in actual patients, suggests that BPE is not predictive for breast cancer in women at increased risk. A possible explanation for this is that in case-control studies, BPE in cancer patients might have been affected by the presence of breast cancer. Consequently, further research into the biological basis and modifying factors of BPE is needed. Alternatively, our results might point to a different carcinogenesis in women at increased risk.

Evidence suggests that BPE correlates negatively with age and increases with hormonal activity [30–32]. Interestingly, our results showed that *BRCA* mutation carriers had significantly lower FGT and BPE values compared to women without a *BRCA* mutation, while the age of *BRCA* mutation carriers was significantly lower than that of women without *BRCA* mutation. This counterintuitive result may be due to differences in the effect of hormones on FGT in women with and without *BRCA* mutation [33]. The fact that we did not observe a difference in BPE between the *BRCA* mutation carriers who did and did not undergo a RRSO before the baseline MRI also points in this direction. Nevertheless, prior research showed that RRSO may still reduce both BPE and FGT values [34], and therefore, our results need to be interpreted with caution as they might also be explained by the relatively low number of women who underwent RRSO in our study. It should be noted that the performance of RRSO in *BRCA* mutation carriers after the baseline examination may have had impact on the predictive value of FGT and BPE in these women.

Women with high BPE scores had a 1.5 times higher chance to get a FPR, and 1.8 times higher chance to get a FPB in the first screening round. This is in line with previous studies, describing that more focal, regional, or asymmetric BPE was associated with a higher likelihood of BI-RADS 3 assessment in the screening setting [35]. Giess et al stated that, in the latter case, it may be hard to distinguish BPE from non-mass enhancement (NME) [36]. Consequently, when the enhancement pattern is interpreted as NME, the reporting radiologist has to consider the possibility of malignancy; thus, chances on false positives increase. DeMartini et al [37] also reported that higher amounts of BPE were associated with higher rates of abnormal interpretation. Brennan et al reported that moderate and marked BPE are associated with significantly higher MR imaging-guided core biopsy cancelation rates compared to minimal or mild BPE [38]. However, the even stronger correlation between BPE and FPB in our study unfortunately suggests that many biopsies are still performed due to BPE. Nevertheless, neither BPE nor FGT is predictive of false-positive recalls or biopsies in subsequent screening rounds, which could mean that BPE and FGT are only affecting false positives when no prior exams are available.

The automated algorithm for BPE estimation eliminates intra- and interrater variability. This is relevant as previous studies reported only a fair interrater variability for BPE when using observer scores according to the BI-RADS lexicon [18]. The automated method provides quantitative measurements and therefore creates an opportunity to define more precise cutoff points. The chosen cutoff was selected based upon previous research, but it is possible that different cutoff points might lead to different results, but did not lead to different conclusions. This is in line with a recent study on the prognostic value of BPE in the contralateral breast of women with unilateral breast cancer, where the effect of different cutoffs appeared to be minimal [39]. Nevertheless, it should be noted that the different methods to assess BPE may also lead to different outcomes in the risk model.

Our study has some limitations. Due to the retrospective nature of our data, it was not always possible to retrieve data on the menstrual cycle or menopausal status. Therefore, we could not correct for these factors. Obviously, menopausal state is partly covered by including age in the risk model. However, future research is needed to better investigate the influence of menopausal status on the predictive value of FGT and BPE for future breast cancer occurrence. In addition, this was a single-institutional study, which potentially limits its generalizability. We chose to include all cancers that were detected in the period after the baseline scan to find a relation between the baseline scan and any future breast cancer occurrence (in on average 3 years follow-up). However, relative risks for a shorter period of time might also be very interesting to study. In addition, changes of FGT and BPE from the baseline might also be predictive of risk. As our cohort is too small to answer these questions, this needs further evaluation in a future study. During the study period, we changed from a 1.5- to a 3-T scanner, and also adapted scanning protocols several times which could potentially influence the results of the BPE calculation algorithm. However, we ensured that BPE was measured between 90 and 120 s after contrast administration, and since BPE is evaluated only semi-quantitatively (in order to imitate the visual inspection by radiologists), we aimed to minimize this effect. Still, further standardization of imaging parameters may improve homogeneity of FGT and BPE estimations and potentially improve the predictive potential. Another possible limitation of the study was that only in the case of false-positive findings did we not exclude women who had a false-positive finding directly after the first screening round. In theory, this could alter FGT and BPE scores, although we minimized this effect by averaging scores over two breasts.

In conclusion, automatically computed FGT and BPE measures at baseline were not associated with subsequent breast cancer occurrence in a cohort of women at high risk for breast cancer. This has implications for personalized screening, as FGT and BPE cannot be implemented in risk prediction models. Higher FGT and BPE were, however, associated with higher rates of false-positive findings at baseline; patient counseling should therefore include these outcomes before starting MRI screening.

## Electronic supplementary material

ESM 1 (DOCX 23 kb)

## Abbreviations

ACR: American College of Radiology
BD: Breast density
BPE: Background parenchymal enhancement
CI: Confidence interval
DCIS: Ductal carcinoma in situ
FGT: Fibroglandular tissue
FPB: False-positive biopsy
FPR: False-positive recall
IQR: Interquartile range
MRI: Magnetic resonance imaging
NME: Non-mass enhancement
OR: Odds ratio
ROC: Receiver operating characteristic
RRSO: Risk-reducing salpingo-oophorectomy
