# Supplementary material for: Amount of fibroglandular tissue FGT and background parenchymal enhancement BPE in relation to breast cancer risk and false positives in a breast MRI screening program: A retrospective cohort study
Source: Eur Radiol. 2019 Feb 22;29(9):4678–90. doi: 10.1007/s00330-019-06020-2 (PMC6682856; doi:10.1007/s00330-019-06020-2)
Supplement: Supplementary file 1 — (DOCX 23 kb) [file 330_2019_6020_MOESM1_ESM.docx]

**Supplementary Table 1.** Risk factor for breast MRI screening

| ***Risk factor*** | ***Number of women (%)*** |
| --- | --- |
| *BRCA* mutation carriers | 573 (37%) |
| Family history, non-*BRCA* | 642 (42%) |
| Other reasons for screening | 318 (21%) |

**Supplementary Table 2.** Background parenchymal enhancement (BPE) values for different cut-off values of the deep-learning system.

|  | **Total cohort**  **(N=1533)** | **Developed cancer**  **Yes (N=60) No (N=1473)** | |
| --- | --- | --- | --- |
| BPE in percentage (median, IQR) (10%) | 67.7 (27.6) | 71.3 (30.4) | 67.6 (27.6) |
| BPE in percentage (median, IQR) (20%) | 50.4 (32.5) | 52.6 (38.8) | 49.8 (32.0) |
| BPE in percentage (median, IQR) (30%) | 38.0 (32.4) | 38.6 (39.7) | 35.3 (31.9) |
| BPE in percentage (median, IQR) (40%) | 29.1 (28.4) | 26.9 (36.1) | 25.6 (28.3) |
| BPE in percentage (median, IQR) (50%) | 22.7 (24.2) | 18.8 (30.4) | 18.6 (23.9) |

IQR: the difference between the 75^th^ and 25^th^ percentiles

**Supplementary Table 3.** Regression coefficients and odds ratios for the different BPE cut-offs (20%, 30%, 40%, 50%) for the effect on current and subsequent MRI scans on cancer false positive findings

| ***Model*** | ***Predictor*** | ***P-value*** | ***Included in final model*** | ***β***  ***(95% CI)*** | ***OR***  ***(95% CI)*** | ***Shrinkage factor*** |
| --- | --- | --- | --- | --- | --- | --- |
| **Prognostic cancer model** | | | | | |  |
| Cancer-BPE  20% - 50% | *BRCA*  Age BPE | 0.001  0.930  ≤0.761 | x  -  - | 1.285 (0.747 – 1.908) | 3.616 (2.110 – 6.743) | 0.96 |
| **Current false positive recall model** | | | | | |  |
| FPR-BPE 20% | *BRCA*  Age  BPE | 0.623  0.120  0.005 | -  -  x | 0.404 (0.133 – 0.704) | 1.498 (1.142 – 2.022) | 0.88 |
| FPR-BPE 30% | *BRCA*  Age  BPE | 0.605  0.144  0.005 | -  -  x | 0.379 (0.098 – 0.665) | 1.461 (1.105 – 1.944) | 0.87 |
| FPR-BPE 40% | *BRCA*  Age  BPE | 0.299  0.046  0.299 | -  x  - | -0.009 (-0.018 – 0.000) | 0.991 (0.982 – 1.000) | 0.70 |
| FPR-BPE 50% | *BRCA*  Age  BPE | 0.299  0.045  0.424 | -  x  - | -0.009 (-0.018 – -0.001) | 0.991 (0.982 – 0.999) | 0.70 |
| **Prognostic false positive recall model** | | | | | |  |
| FPR-BPE 20% - 50% | *BRCA*  Age  BPE | 0.773 0.001 ≤0.839 | - x - | -0.047 (-0.070 – 0.027) | 0.954 (0.932 – 0.973) | 0.95 |

For every model different shrinkage factors were used, shrunk β and OR are presented.
* FGT and BPE = low is reference category
β: standardized coefficients, OR: odds ratio, CI: confidence interval, FGT: amount of fibroglandular tissue, BPE: background parenchymal enhancement
